# Supplementary material for: A Spatial Quantitative Systems Pharmacology Platform spQSP-IO for Simulations of Tumor–Immune Interactions and Effects of Checkpoint Inhibitor Immunotherapy
Source: Cancers (Basel). 2021 Jul 26;13(15):3751. doi: 10.3390/cancers13153751 (PMC8345161; doi:10.3390/cancers13153751)
Supplement: Supplementary file 1 [file cancers-13-03751-s001.zip › supplement/cancers-1225495-supplementary_GC_2.pdf]

# Supplementary Materials: A Spatial Quantitative Systems Pharmacology Platform spQSP-IO for Simulations of Tumor–Immune Interactions and Effects of Checkpoint Inhibitor Immunotherapy

**Chang Gong, Alvaro Ruiz-Martinez, Holly Kimko and Aleksander S. Popel**

## Text S1

*Changes in the QSP Module Equations from the Original Model of Jafarnejad et al [1]*

In the hybrid spQSP-IO model, the ODE-based QSP module capturing the whole-system dynamics is imported from Jafarnejad et al [1]. For compatibility with the spQSP-IO platform and consistency with more recent studies published on QSP-IO platform [2], we made changes in the QSP module, and SBML of the modified model is provided (Supplement_QSP_IO_NSCLC_SBML.xml). Specifically, changes to the original model include:

1. Weight factor added to species and rates in order to reflect partial representation of the tumor compartment in the ODE system. This is added post SBML export, so the QSP module equations do not reflect this change. However, this modification is included in the configuration file (Supplement_QSP_IO_config.xml) for the SBML model conversion software (links to Github).
2. Two new parameters allowing separate control of the death rate of exhausted CD8 Teff in the tumor compartment (Parameter 173) and IL-2 induced Treg proliferation in the LN (Parameter 174).
3. Procedure of initialization of the ODE system is changed. Previously, the whole system was simulated for one year with cancer cell number hold constant so that the system reached steady state, and the resulting values of each species were used as initial condition. In the current version, initial number of cancer cells is set to twice the minimum allowed value and simulation continues until the tumor grows to designated initial volume, then the simulation continues as time is set to 0.
4. Proliferation rate of activated T cells is no longer proportional to the inverse of the expected number of rounds of division, as in Reaction 55:

$$\frac{dLN.Teff\_1\_0}{dt}|_{prolif} = k\_aT\_prolif * 2^{n\_at\_prolif} * LN.aT\_1\_0$$

5. Adhesion rate proportional to T cell concentration, rather than number of T cells in the tumor vasculature. Reaction 59 represents the adhesion and transmigration of Teff to tumor from blood and is shown below. Reaction 75 is the adhesion and transmigration of Tregs, which is similarly modified (not shown).

$$\frac{dTum.Teff\_1\_0}{dt}|_{adhision} = k\_Teff\_transmig * S\_adhesion\_tot * \frac{Tum * f\_vol\_BV * Tum.C1}{K\_C\_max} * \frac{Cent.Teff\_1\_0}{Cent} * Tum$$

*Number of Roots for PDL1-PD1 Bounds (Equation 3)*

I If $T_2 = 0$:

$$PDL1\_syn = 0, PD1\_PDL1 = 0$$

II If $T_2 \neq 0$:

$$PDL1\_syn \neq 0, \quad PD1\_PDL1 \neq 0$$

Denote $PD1\_PDL1 = xT_2, \quad 0 < x < 1$

$$x^3 - \left(2 + \frac{1}{k_1T_2} + \frac{T_1}{T_2} + \frac{Nivo * k_2}{k_1T_2}\left(1 - \frac{2k_3}{k_1}\right)\right)x^2 + \left(1 + \frac{1}{k_1T_2} + \frac{2T_1}{T_2} + \frac{Nivo * k_2}{k_1T_2}\right)x - \frac{T_1}{T_2} = 0 \ (1)$$

II.a. If $Nivo = 0$:

$$x^3 - \left(2 + \frac{1}{k_1T_2} + \frac{T_1}{T_2}\right)x^2 + \left(1 + \frac{1}{k_1T_2} + \frac{2T_1}{T_2}\right)x - \frac{T_1}{T_2} = 0$$

$$(x-1)\left(x^2 - \left(1 + \frac{1}{k_1T_2} + \frac{T_1}{T_2}\right)x + \frac{T_1}{T_2}\right) = 0$$

$$x^2 - \left(1 + \frac{1}{k_1T_2} + \frac{T_1}{T_2}\right)x + \frac{T_1}{T_2} = 0 \ (2)$$

$$\Delta = \left(1 + \frac{1}{k_1T_2} + \frac{T_1}{T_2}\right)^2 - \frac{4T_1}{T_2} > \left(1 + \frac{T_1}{T_2}\right)^2 - \frac{4T_1}{T_2} = \left(1 - \frac{T_1}{T_2}\right)^2 \geq 0$$

Thus (2) has two distinct roots $x_1$, $x_2$, and $x_1 < x_2$

$$x_1 + x_2 = 1 + \frac{1}{k_1T_2} + \frac{T_1}{T_2}$$

$$x_1x_2 = \frac{T_1}{T_2}$$

$$x_1 + x_2 = 1 + \frac{1}{k_1T_2} + x_1x_2 > 1 + x_1x_2$$

$$x_1x_2 - x_1 - x_2 + 1 < 0$$

$$(x_1 - 1)(x_2 - 1) < 0$$

$$0 < x_1 < 1, \quad x_2 > 1$$

$$x = x_1 = \frac{1}{2}\left(\left(1 + \frac{1}{k_1T_2} + \frac{T_1}{T_2}\right) - \sqrt{\Delta}\right)$$

II.b. If $Nivo \neq 0$:

Let

$$f(x) = x^3 - \left(2 + \frac{1}{k_1T_2} + \frac{T_1}{T_2} + \frac{Nivo * k_2}{k_1T_2}\left(1 - \frac{2k_3}{k_1}\right)\right)x^2 + \left(1 + \frac{1}{k_1T_2} + \frac{2T_1}{T_2} + \frac{Nivo * k_2}{k_1T_2}\right)x - \frac{T_1}{T_2}$$

$$f(0) = -\frac{T_1}{T_2} < 0$$

$$f(1) = \frac{2Nivo * k_2k_3}{k_1^2T_2} > 0$$

Thus there must exist at least one real root in (0, 1). Assuming there are more than 1 real roots (including multiple roots) in (0, 1),

Because f(0) < 0, f(1) > 1, the three roots, $x_a$, $x_b$, $x_c$ must be all in (0, 1)

Let

$$0 < \mathrm{x_a} \leq \mathrm{x_b} \leq \mathrm{xc} < 1,$$

$$x_a x_b x_c = \frac{T_1}{T_2} > 0$$

$$x_a x_b + x_b x_c + x_c x_a = 1 + \frac{1}{k_1 T_2} + \frac{2T_1}{T_2} + \frac{Nivo * k_2}{k_1 T_2} > 1 + \frac{2T_1}{T_2} = 1 + 2x_a x_b x_c$$

$$\therefore x_a x_b + x_b x_c + x_c x_a - 2x_a x_b x_c > 1$$

$$\therefore x_a(x_b + x_c - 2x_b x_c) > 1 - x_b x_c$$

$$x_b + x_c - 2x_b x_c > 2x_b - 2x_b x_c = 2x_b(1 - x_c) > 0$$

$$\therefore x_b \geq x_a > \frac{1 - x_b x_c}{(x_b + x_c - 2x_b x_c)}$$

$$\therefore x_b + x_c - 2x_b x_c > \frac{1}{x_b} - x_c$$

$$\therefore 2x_c(1 - x_b) > \frac{1 - x_b^2}{x_b}$$

$$\therefore x_c > \frac{1 + x_b}{2x_b} > 1$$

This contradicts $x_c < 1$.

Thus, there is one and only one root in (0, 1).

**Text S2**

*The Role of Maximum Number of Divisions for Cancer Cells In Tumor Morphology*

In order to examine the impact of the unlimited division potential (or the lack of it) of CSCs on the shape of the tumor, we performed two set of additional simulations: 1. Simulations with cancer progenitor cells only, and 2. Simulations with the original settings, while varying the number of max divisions for progenitor cells. For simulation set 1, adjustment to initial conditions and parameter values are made for this set of simulations compared to the ones where CSCs are included. In the initial condition, all initial cancer cells are progenitors (CSC fraction set to 0). Division rate for progenitor cells is set based on tumor growth rate, which is lower than the original division rate of progenitors. Movement probability of cancer progenitor cells is also lowered to compensate for the reduced density of cells in the tumor. The maximum number of divisions for progenitor cells is set to 5, 10 and 20. For simulation set 2, we choose the combination of three maximum progenitor division number and two asymmetric division probabilities and two movement probabilities (a total of 12 scenarios). The results are shown in Figure S1 and Figure S2.

From Figure S1, we can see that without CSC, the tumor resulting from the simulation is approximately spherical, regardless of movement probability of cancer cells. When the number of maximum divisions is small, the tumor disappears when all division potential from progenitor cells is exhausted (rows 1–3). When the maximum number of divisions is high, the morphology does not change with this parameter at the corresponding time points.

From Figure S2, we can see that more invasive tumor morphology is associated with higher movement probability of cancer cell (columns 4–6) and lower asymmetric division probabilities. The impact of maximum number of divisions on morphology is two-fold: on the one hand, smaller number of divisions result in more disseminated pattern of cancer cells; on the other hand, larger number of divisions result in more cancer cells and larger tumors, with clearer finger-like protrusions.

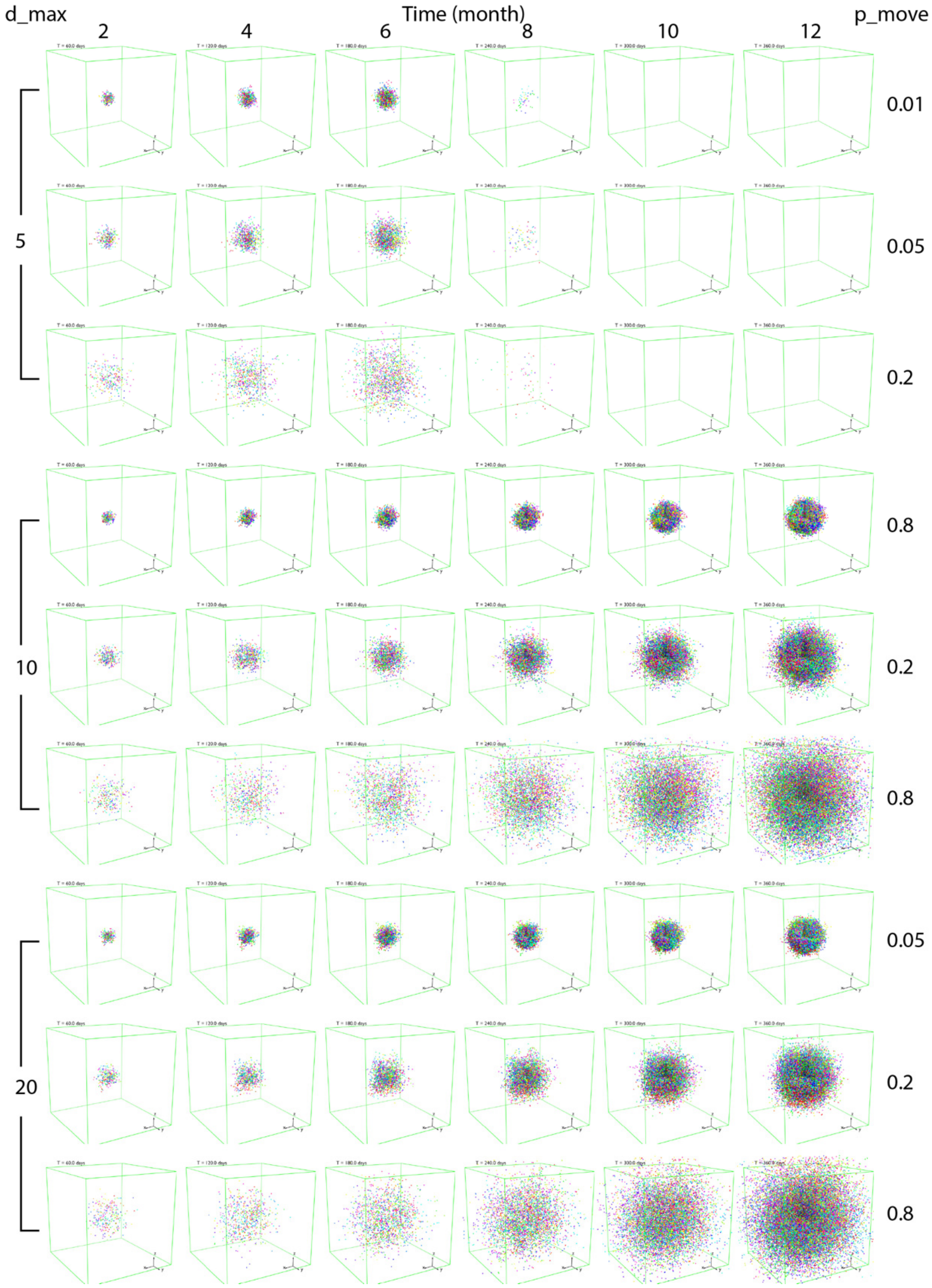


**Figure S1.** Simulation of tumor growth with no CSC. Colors represent lineages of original progenitor cell.

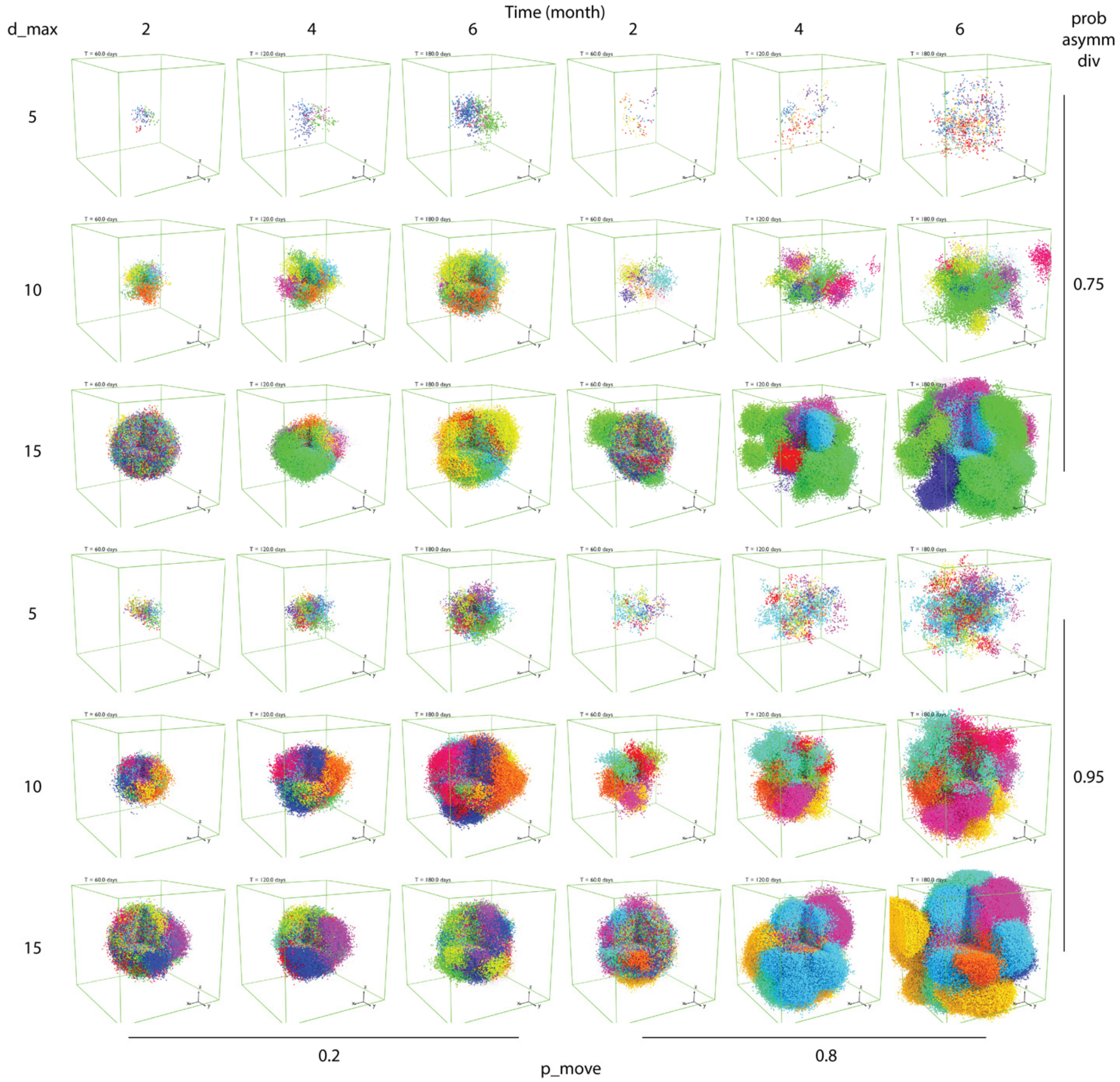


**Figure S2.** Simulation of tumor growth with CSC and varying maximum progenitor division numbers. Colors represent the lineage of CSC from which the progenitor cells are derived.

*Virtual Patients Responsiveness to Nivolumab Treatment ($W_{QSP}$ = 1)*

In order to ensure that the results from the previous stand-alone QSP model are reproducible with the hybrid model based on the current spQSP-IO platform with the modified QSP, we performed the simulations using data from a clinical trial (ClinicalTrials.gov number NCT02259621) [3] with the same setup as in our previous study [1]. Two parameters, mutational burden and antigen strength, are assigned to 12 virtual patients based on measurements from the clinical trial, while other parameters are varied for each of these patients to account for uncertainties from unmeasured characteristics. The QSP module parameters are the same as used in the original study, while the weight of QSP module $w_{QSP}$ is set to 1. Three arms of treatments are simulated: no treatment (blue), biweekly nivolumab of 3 mg/kg for a year (red), and two doses of 3 mg/kg nivolumab infusion followed by resection (blue). Results are shown in Figure S1. Solid lines represent median values at each point, while the shadowed areas represent a 60% pointwise prediction intervals.

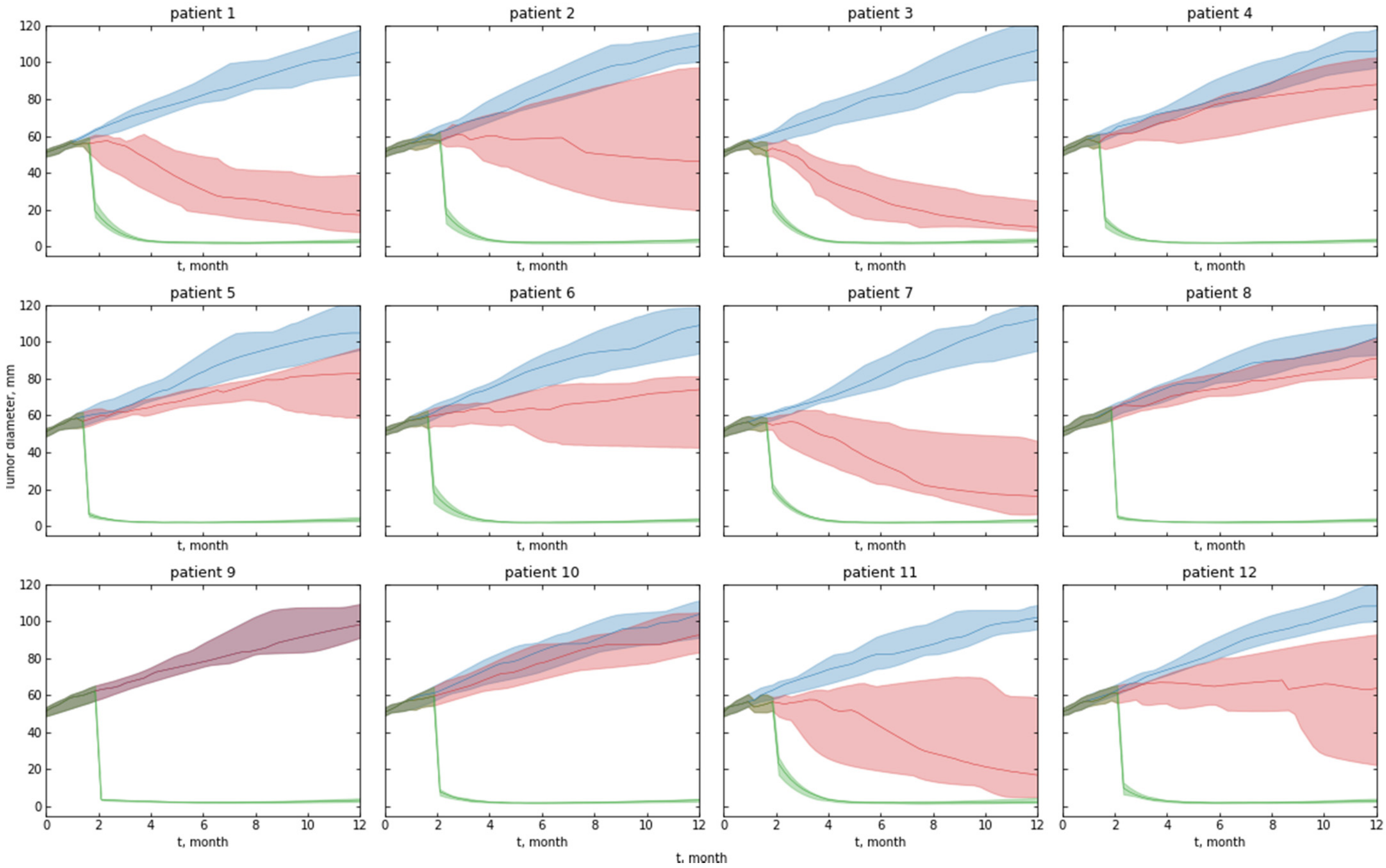


**Figure S3.** Tumor diameter dynamics with 12 virtual patients. Blue: untreated. Red: neo-adjuvant nivolumab treatment. Green: nivolumab + resection.

*Full Temporal Dynamics of QSP/ABM Modules*

Simulation outputs from the virtual cohort are shown in Figures S4–S7. In all plots, each time course represents one virtual patient, with color representing tumor growth rate for each simulation (yellow: highest tumor growth rate; purple: lowest tumor growth rate). 100 Patients are randomly selected from the entire virtual population of 1000 so that individual lines are visible.

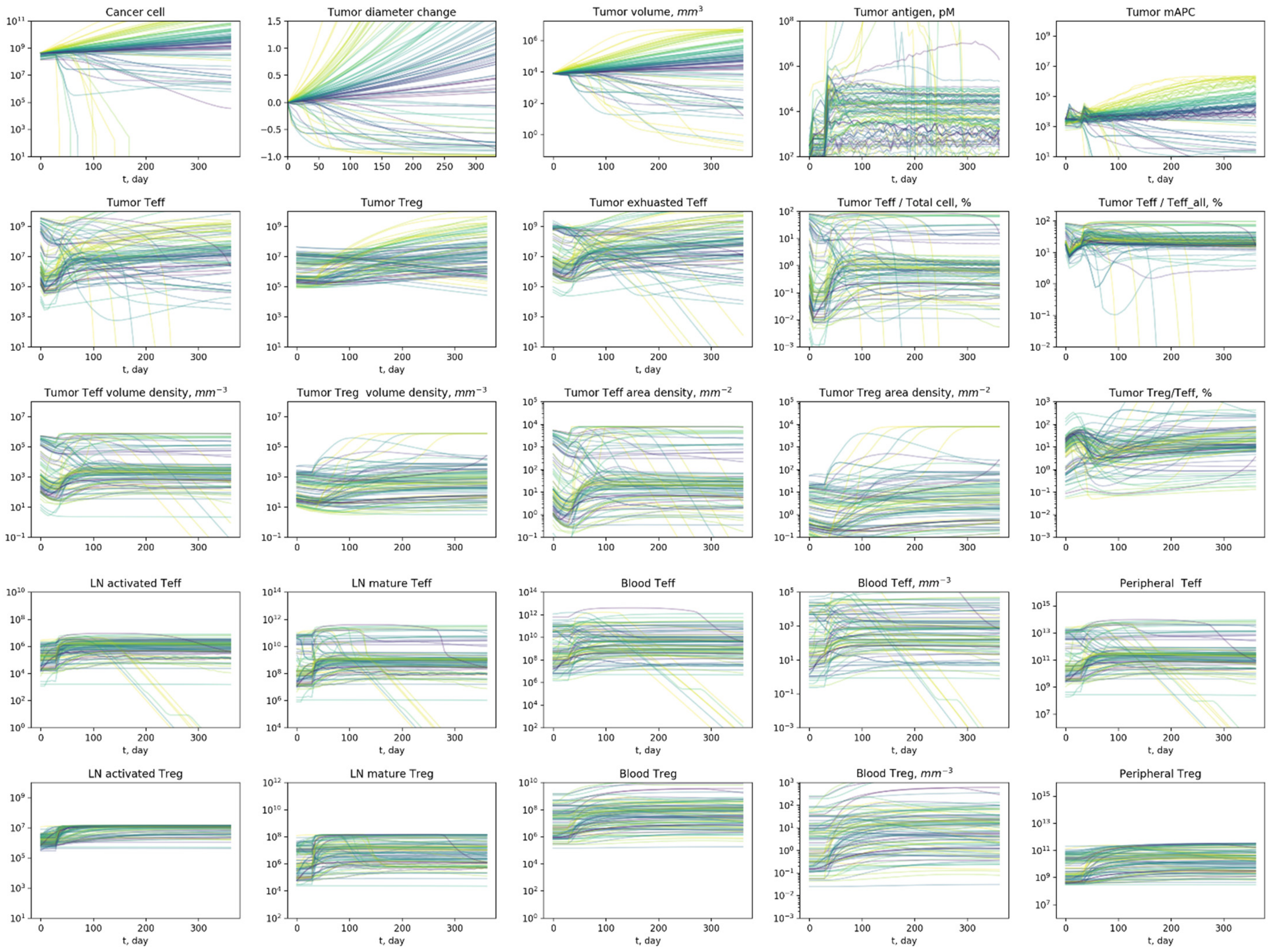


**Figure S4.** Dynamics of overall tumor growth and number of T cells in various compartments.

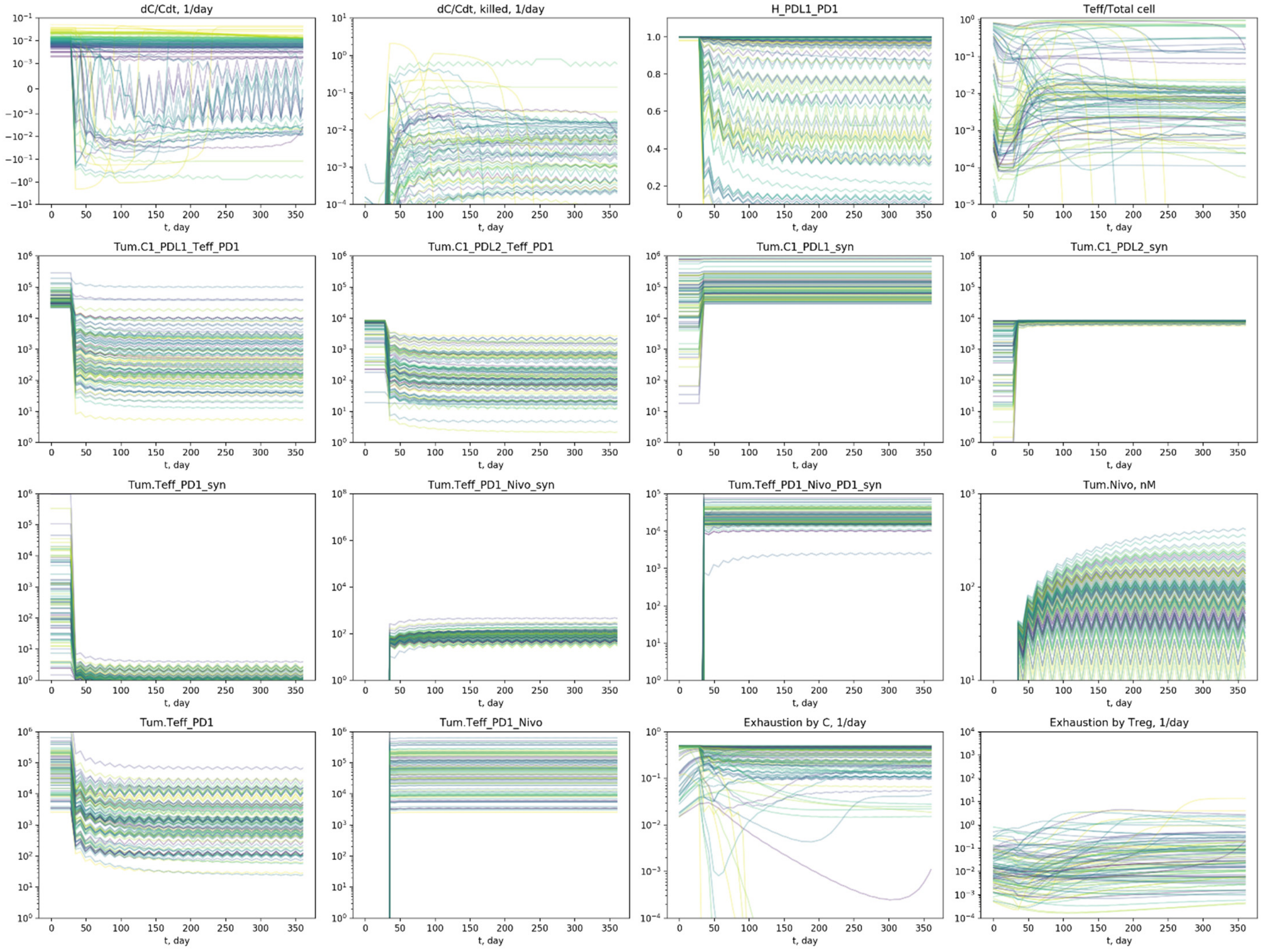


**Figure S5.** Dynamics related to cancer cell killing by Teff.

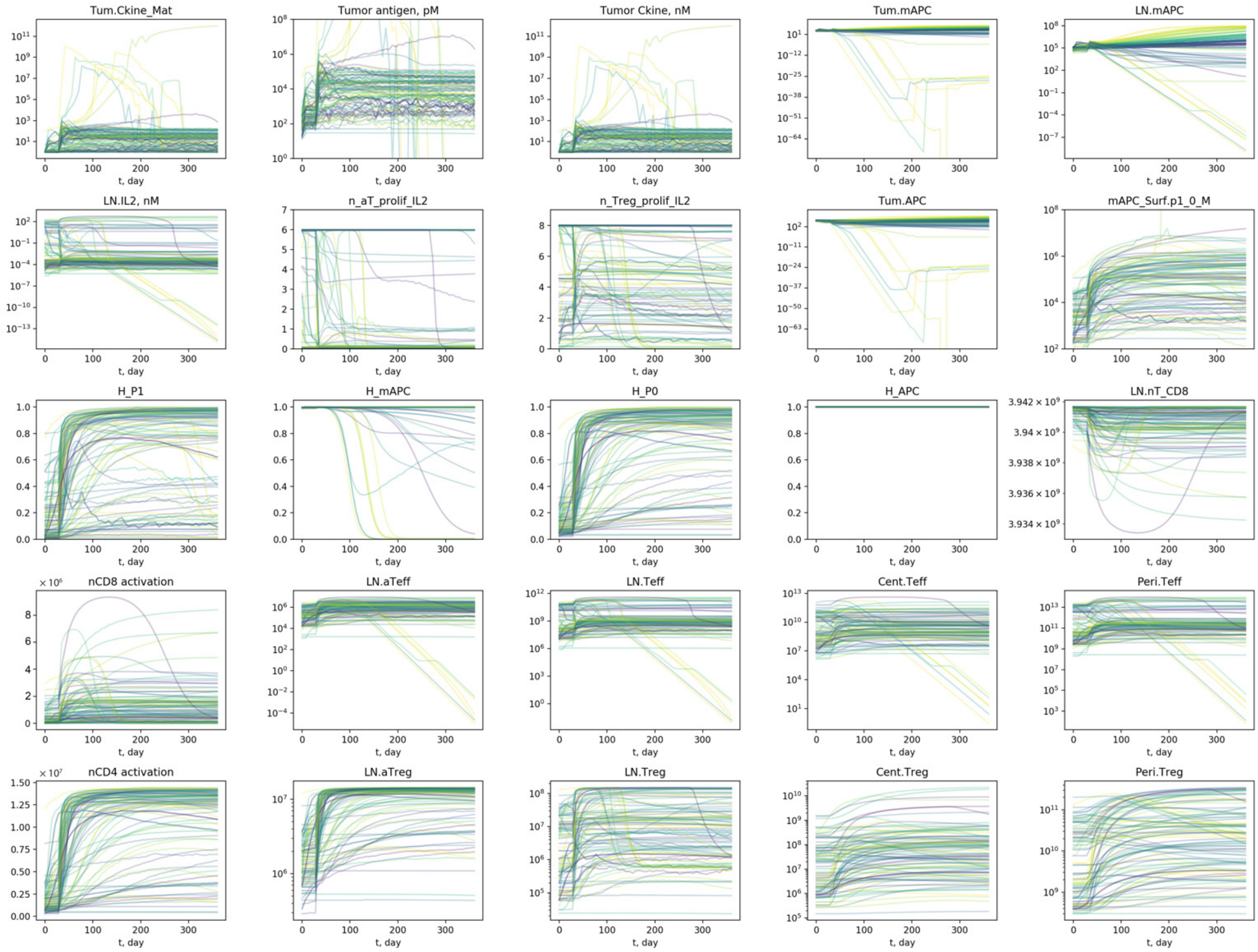


**Figure S6.** Dynamics related to T cell priming.

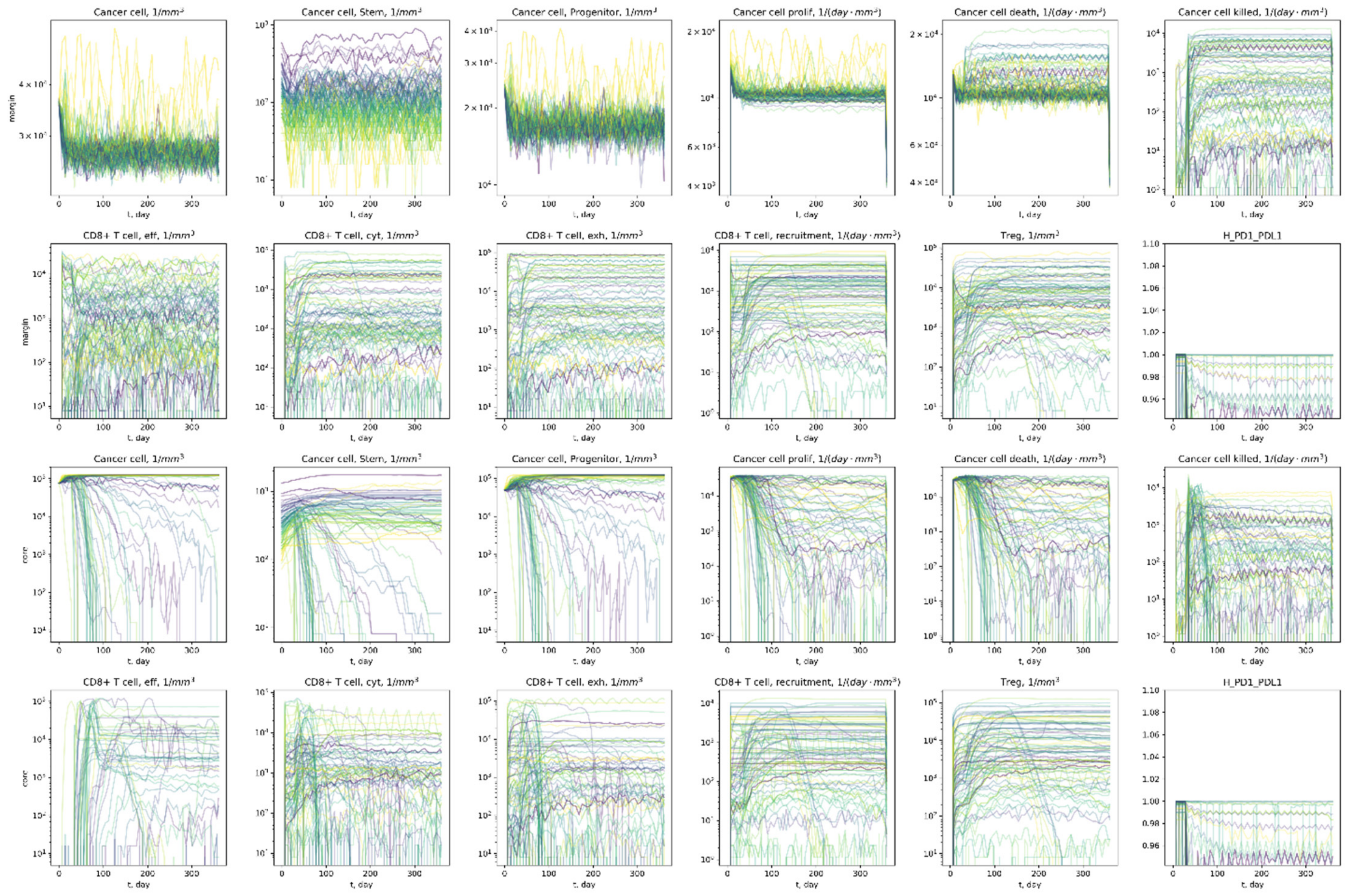


**Figure S7.** ABM dynamics in the invasive front and tumor core ROIs.

## Text S3

*Biomarker Analysis*

The procedure (forward selection) to identify biomarkers from candidates can be represented in the following pseudo code:

A = {}

U = {1, 2, …, n} # 1, 2, …, n are indices of biomarker candidates

# $A^C$ is the set of biomarkers not included in A

while $A^C \neq \emptyset$:

  for each $j \in A^C$:

    # fit regression model with one extra candidate

   # biomarker plus the current set A biomarkers

    fit (Y, [$X_j$, $X_l$], $l \in A$) to model

    calculate LRT p-value pj

  identify j* to minimize pj and pj < $\alpha$

  if  $\exists$ j*:

    add j to A

  else:

    break

In the resampling process, the number of times each biomarker is selected out of 100 evaluations is listed in Table S1. The biomarkers selected more than half of the time (in bold) are chosen for further analysis.

**Table S1.** Number of times biomarker candidates chosen in resampling.

| Biomarker | Tumor Diameter | ORR | Time to Progression |
|---|---|---|---|
| Teff, Blood | 17 | **66** | **81** |
| Treg, Blood | 19 | **54** | 39 |
| Treg-Teff ratio, blood | 5 | 19 | 32 |
| Teff, Tumor | 25 | **58** | 37 |
| Treg, Tumor | 13 | 30 | 8 |
| Treg-Teff ratio, tumor | 11 | 25 | 17 |
| Cancer cell, Tumor | **98** | 21 | **57** |
| Tumor diameter | **100** | 7 | **70** |
| Teff IF | 11 | 11 | 13 |
| Treg, IF | 45 | 19 | 40 |
| Treg-Teff ratio, IF | 1 | 10 | 8 |
| PDL1, IF | 23 | 45 | 32 |
| Cancer cell, IF | 36 | 14 | **65** |
| Teff, Core | 21 | 25 | 28 |
| Treg, Core | 21 | 7 | 15 |
| Treg-Teff ratio, Core | 11 | 21 | 23 |
| PDL1, Core | 2 | 16 | 9 |
| Cancer cell, Core | **93** | **95** | **98** |

*Table of Abbreviations*

**Table S2.** Table of Abbreviations.

| Abbreviation | Full Name |
|---|---|
| QSP | Quantitative systems pharmacology |
| ODE | Ordinary differential equation |
| PDE | Partial differential equation |
| ABM | Agent-based models |
| PK/PD | Pharmacokinetic/Pharmacodynamic |
| LHS | Latin Hypercube Sampling |
| PRCC | Partial Rank Correlation Coefficient |
| LRT | Likelihood ratio test |
| SBML | Systems biology markup language |
| NSCLC | Non-small cell lung cancer |
| LN | Lymph node |
| MDSC | Myeloid-derived suppressor cells |
| CSC | Cancer stem-like cell |
| APC | Antigen-presenting cells |
| ITH | Intratumoral heterogeneity |
| TMA | Tissue microarray |

**Supplement_CSC** is provided separately attached as an .mp4 file.

**Supplement_params, Supplement_QSP_IO_config,** and **Supplement_QSP_IO_NSCLC_SBML** are provided separately attached as .xml files.

**Supplement_QSP_IO_MODEL** is provided separately attached as an .xls file.

## References

1. Jafarnejad, M.; Gong, C.; Gabrielson, E.; Bartelink, I.H.; Vicini, P.; Wang, B.; Narwal, R.; Roskos, L.; Popel, A. A Computational Model of Neoadjuvant PD-1 Inhibition in Non-Small Cell Lung Cancer. *AAPS J.* **2019**, *21*, 1–14, doi:10.1208/s12248-019-0350-x.
2. Sové, R.J.; Jafarnejad, M.; Zhao, C.; Wang, H.; Ma, H.; Popel, A.S. QSP-IO: A Quantitative Systems Pharmacology Toolbox for Mechanistic Multiscale Modeling for Immuno-Oncology Applications. *CPT: Pharmacometrics Syst. Pharmacol.* **2020**, *9*, 484–497, doi:10.1002/psp4.12546.
3. Forde, P.M.; Chaft, J.E.; Smith, K.N.; Anagnostou, V.; Cottrell, T.R.; Hellmann, M.D.; Zahurak, M.; Yang, S.C.; Jones, D.R.; Broderick, S.; et al. Neoadjuvant PD-1 Blockade in Resectable Lung Cancer. *New Engl. J. Med.* **2018**, *378*, 1976–1986, doi:10.1056/nejmoa1716078.
